# Cultural differences in attitudes towards surgical site infections among French anesthetists and surgeons in digestive surgery in 2022

**DOI:** 10.1186/s13756-025-01576-9

**Published:** 2025-05-28

**Authors:** Antoine Deslandes, Niki Christou, Patrice Baillet, Joseph Hajjar, Philippe Marre, Hubert Johanet, Marc Leone, Gabriel Birgand

**Affiliations:** 1 Regional Center for Infection Prevention and Control (CPias), Region of Pays de la Loire, Nantes University Hospital, 5 rue du Pr Yves Bocquien, Nantes, 44000 France; 2 Académie Nationale de Chirurgie, Paris, France; 3 Association Française de Chirurgie (AFC), Paris, France; 4 Digestive Surgery Department, University Hospital Limoges, Limoges, France; 5 Société Française d’Anesthésie Réanimation (SFAR), Paris, France; 6 Anesthesia and Intensive Care Medicine, Hôpital Nord, Assistance Publique Hôpitaux de Marseille, Aix Marseille Université, Marseille, France; 7 Cibles et médicaments des infections et de l’immunité, IICiMed, Nantes Université, UR 1155, Nantes Université, Nantes, France; 8 National Institute for Health and Care Research (NIHR) Health Protection Research Unit in Healthcare Associated Infections and Antimicrobial Resistance at Imperial College London, London, UK

**Keywords:** Antimicrobial resistance, Quality and safety, Surgery, Digestive surgery, Surgical site infection, Infection prevention and control, Qualitative study

## Abstract

**Background:**

Although it generates a significant burden, little attention has been paid to preventing Surgical Site Infection (SSI) in digestive surgery.

**Objective:**

This study explored the factors underpinning anesthetists’ and surgeons’ attitudes toward SSI prevention in digestive surgery, focusing on their perceptions of SSI, preventive measures, guidelines, and cooperation across both specialties.

**Methods:**

Qualitative semi-structured interviews were conducted with 15 surgeons and 19 anesthetists working in digestive surgery. Participants were approached through established mailing lists and snowball sampling. Interviews were recorded and transcribed verbatim. Transcripts were coded and analyzed thematically using a constant comparative approach.

**Results:**

SSI in digestive surgery was perceived as an inevitable consequence and ranked down in the priorities of surgeons. A paradox existed between the low consideration of superficial infections that are easily manageable through antibiotics and the strong awareness of the antibiotic resistance threat. Global trust appeared regarding the guidelines, but a knowledge gap of the guidelines was observed among surgeons in comparison with anesthetists. SSI ownership was perceived as collective, but the responsibility belonged to the surgeon alone. Surgeons focused on actions and short-term tasks within a culture of individualism, whereas anesthetists worked collectively with systemic approaches. Overall, the cooperation between both specialties was positive, but tightly reliant on teamwork, workload, and organization in the operating theatre.

**Conclusions:**

The cultural differences between surgeons and anesthetists should be recognized as a key overarching factor in defining their respective roles in the prevention of SSI and in establishing accountability in digestive surgery — including aspects such as adherence to guidelines, and the implementation of preventive measures.

**Supplementary Information:**

The online version contains supplementary material available at 10.1186/s13756-025-01576-9.

## Introduction

Surgical site infections (SSI) are among the most frequent healthcare-associated infections (HAI), accounting for approximately 20% of all HAI in hospitalized patients in Europe [1]. Digestive surgery is associated with a high SSI burden, accounting for 38% of all SSI across specialties [2]. Despite this significant burden, SSI prevention in this specialty does not seem to be a top priority, as opposed to clean surgeries [3]. This paradox can be related to the perceived inevitability of SSI in this surgery due to the contamination of the surgical site by digestive content, accessible treatment of the infection, and overall lower culture prevention [4].

Although there are multiple national and international guidelines for the prevention of SSI [5–7], compliance with best practices still requires improvement. For example, compliance with best practices for surgical antibiotic prophylaxis (SAP) remains suboptimal, with 59% adherence to SAP guidelines in the US [8]. In France, 64% adherence to SAP guidelines was described, with a lower rate of digestive surgery and compliance with SAP for colorectal cancer surgeryat 44% [9]. This lack of compliance may come from the guidelines themselves [10], including the heterogeneity in content, level of evidence [11], or methods used to develop and write recommendations [12].

Infection prevention and control (IPC) strategies tend to target individual factors such as knowledge, motivation, and skills, leaving social and organizational determinants on the side lines despite their impact on adherence to guidelines [10]. Nevertheless, other factors such as hierarchical relations in operating rooms [13], attitude toward infection prevention measures, and accountability for SSI are of critical importance. Anesthetists and surgeons are both accountable for the quality of healthcare and security of their patients, including some SSI preventive measures (e.g., SAP, normothermia), with good cooperation. Working dynamics and hierarchy across these two specialties are key to ensuring effective perioperative care. However, the suboptimal implementation of SAP illustrates the difficulties of collaboration [14]. In-depth studies of the social determinants and roles of surgical and anesthetist practices are needed to explore individual and collective factors underpinning SSI prevention practices and to accurately implement interventions to prevent SSI in digestive surgery [15, 16].

This study aimed to explore the perception of SSI events, knowledge, representation, and use of SSI prevention guidelines, and the respective roles and cooperation of medical actors in SSI prevention among anesthetists and surgeons in digestive surgery.

## Methods

### Setting and study design

This study was conducted on a national scale on behalf of a study group at the French National Academy of Surgery. We designed a qualitative study based on semi-structured interviews with a panel of surgeons and anesthetists working in digestive surgery to describe SSI perception, cooperation, and adherence to SSI prevention guidelines. An interview guide for semi-structured interviews was developed in collaboration with experts involved in intra-and perioperative SSI prevention (Table 1).

**Table 1:** Interview guide, including supplementary questions

Context	Can you describe your role/specialty for the record? In which region do you practice? In what type of facility? (public/private, university hospital…)? How many years of experience do you have in this position?
Risk perception and role in the prevention of SSIs	• How important do you consider SSIs? o What are the consequences for your patients? • To what extent do you think SSIs constitute an important issue in France? o In your institution? In your specialty? • Which aspects of SSI prevention are you involved in? • Is it easy or difficult to comply with SSI prevention? Why? o What factors are associated with the occurrence of SSI? • In your department, where do the responsibilities lie in terms of SSI prevention? And in terms of SSI occurrence? Do you think this point is clear within your organization?
Knowledge of SSI prevention recommendations Barriers and facilitators to adherence to the application of recommendations	• Are you aware of specific SSI prevention frameworks? • Are you aware of a local SSI prevention framework applied in your facility? o Is it easy or difficult to implement SSI prevention measures? Why? • What do you think are the barriers to implementing these recommendations? • How much confidence do you have in the current SSI prevention policy? Do you apply it daily? • Do your colleagues adhere to the policy? • What personal challenges do you face when optimizing your SSI prevention practices? • What would help you in SSI prevention?
Cooperation between surgeon and anesthetist	• What do you consider important in terms of the role of your colleagues in preventing HAIs (Healthcare-Associated Infections)? Do you think the relationships between surgeons and anesthetists influence the prevention of HAIs? Why? • In your experience, how would you describe the cooperation between surgeons and anesthetists in preventing HAIs? What do you think about this cooperation during the pre-operative phase? And in the postoperative phase? • Does the context of the operation (emergency or scheduled, staffing…) impact your ability to cooperate to prevent HAIs? • In your opinion, what could improve cooperation between surgeons and anesthetists and help reduce HAIs?
End	• Is there anything important you’d like to add that hasn’t been addressed on the subject today?

Abbreviations: HAI, healthcare associated infections; SSI, surgical site infection

### Inclusion criteria and recruitment

To be eligible for the study, participants had to work in digestive surgery as senior surgeons or anesthetists with a primary working position in France. We aimed to have participants representative of various sectors [17] and to rely on an equal number of surgeons and anesthetists. A total of 40 participants, including 20 surgeons and 20 anesthetists, were anticipated, including 16 participants working in public hospitals; four participants working in private non-profit hospitals, and 20 participants working in private for-profit hospitals.

### Qualitative semi-structured interview methodology

Between May 30th and September 20th 2022, 64 practitioners (35 surgeons and 29 anesthetists) were invited to participate in this study. The research team contacted the participants through the distribution lists of the national societies of anesthesia and surgery. Contact between the research team and potential participants occurred through e-mail. Reminders were sent after two and four weeks. All participants who responded to this invitation were invited to a face-to-face or videoconferencing interview at a time convenient for them. All participants received an information letter before their interviews. Face-to-face interviews were conducted by AD (Medical Doctor in Public Health performing a Master’s degree in epidemiology) in a private location. Semi-structured interviews were anticipated to last for 20–40 min. The participants were recruited until theme saturation was reached. Video interviews were recorded using Zoom v5.10.4, while face-to-face interviews were conducted using Audacity v3.1.3. The analysis was performed alongside the data collection. Audio and video files were deleted once transcripts were available. The anonymized transcripts were stored on a dedicated server. Data and relevant documents were stored for a minimum of ten years after the end of the study, including the follow-up period.

### Data analysis

Recorded interviews were transcribed into text, and the data were coded using the qualitative data analysis software Qualcoder 3.1. Data were analyzed through thematic analysis [18] using both deductive and inductive coding, relying on a codebook developed based on the interview guide. The analysis was performed by one investigator (AD) after analyzing the first transcripts for coding adequacy and consistency. The data were then reviewed by a second investigator (GB) to set up themes, sub-themes, and hierarchies between the themes. The supporting quotes were translated into English by the investigator. (original cited quotes in Supplementary Material, Table 2).

**Table 2:** Participant characteristics for the semi-structured interviews

	*n* (%) *N* = 34
**Occupation**	
Anesthesiology	19 (56)
Surgery	15 (44)
**Gender**	
Male	17 (50)
Female	17 (50)
**Experience** (year, mean, min-max)	16 (3–33)
**Institution**	
Private, for profit hospital	16 (47)
Private, not for profit hospital	4 (12)
Public sector	14 (41)
**Interview type**	
Face to face	4 (12)
Videoconference	30 (88)

## Results

Of the 64 practitioners working in digestive surgery invited to the semi-structured interviews, 37 (57.8%) initially agreed to participate and 34 (53.1%) were interviewed (3 did not answered to follow-up e-mails). Among them, 19 (56%) were anesthetists (11 [58%] female) and 15 (44%) were surgeons (6 [40%] female). The majority (20, 59%) worked in private facilities, and 14 (41%) worked in public hospitals. (Table 2, Supplementary Table 3) Interviews were mainly performed via videoconferencing (30, 88%), with a mean duration of 32 min (Min: 16; Max: 53 min).

### Preventing a somewhat inevitable consequence is not the priority

Most participants considered SSI to be of paramount importance in digestive surgery (Table 3, Q1-2), related to both the consequences of such an infection for patients and the patient’s perception of surgery.

**Table 3:** Interviews themes and subthemes

Theme	Sub-theme	Illustrative quotations
Preventing a somewhat inevitable consequence is not the priority	Careful consideration of the SSI risk	Q1. *Ah well*,* when you do digestive surgery*,* it’s a major problem because it’s one of the… One of the major postoperative problems in this surgery. […] No*,* no*,* it’s an integral part of the specialty* {Subject 11, Surgeon}
	Severity of SSI and consequences for patients	Q2. *So the consequences range from the simple to the very complicated*,* knowing that the very complicated is often very heavy in this specialty*,* so there you go…* {Subject 11, Surgeon} Q3. *One patient out of 5*,* will present a surgical site infection*,* which is a problem that will lead to excess morbidity*,* that will lead to a longer hospital stay*,* that will lead to sequelae at the level of the wall with a risk of eventration*, etc.,* a delay in convalescence*,* and a cost to society* {Subject 16, Surgeon}
	Singularity of digestive surgery	Q4. *Since we operate on an organ that is full of microbes*,* surgical site infections are major and are more important than in other specialties such as orthopaedic surgery or neurosurgery.* {Subject 16, Surgeon}
	SSI as an inevitable event	Q5. *We are working in a septic environment*,* and some infections occur even if the measures are respected. We are working on the digestive tract which is kind of a “lame” organ*,* it tends to spill its contents quite easily and create infections*. {Subject 7, Anaesthetist}
	Association between SSI and antimicrobial resistance	Q6. *Very clearly*,* in France*,* we see a context of overuse of antibiotics*,* the emergence of resistant bacteria*,* and the need to use these antibiotics to prevent surgical site infections*,* so it is very important for me*,* in the French context*,* to have help in implementing appropriate surgical antibiotic prophylaxis during my anaesthetic procedures*. {Subject 32, Anaesthetist}
	Distinction between superficial and deep SSI	Q7. *So digestive and visceral surgery is very broad*,* we have to separate two situations*,* well several situations that are at risk: there is one that is similar to that of orthopaedics*,* that is wall surgery*,* where in fact we do not normally have contamination of the surgical site*,* but with the implantation of a prosthesis the infectious risk… it’s not that it is increased*,* it’s that it is more difficult to manage* {Subject 14, Surgeon}
	Conflicting priorities	Q8. *I think that under the mass of work*,* the rhythm of the work… In the end it is kind of omitted if you know what i mean […] Too many other things to think about.* {Subject 10, Anaesthetist}
	Ignorance of SSI rates	Q9. *I think that*,* at least what we are given as information*,* which we do not have*,* it is regrettable*,* it would be good if we could have in fact*,* almost in real time the data on these surgical site infections* {Subject 18, Surgeon}
A global trust in guidelines but an apparent knowledge gap	Trust in SSI guidelines and guidelines producers	Q10. *No*,* well*,* I have complete confidence in the guidelines*,* they are… They’re going to… I have confidence in my professional society and in the recommendations that it puts out* {Subject 30, Anaesthetist}
	Easy use of guidelines	Q11. *It doesn’t seem to me insurmountable*,* it’s preparation*,* it’s established protocols*,* after that it’s to be a little careful… […] so it doesn’t seem that complicated to me.* {Subject 22, Surgeon}
	Key role of the surgical safety check list	Q12. *And then we have to discuss*,* so this is not bad*,* it is a… It’s not too bad in my case*,* but there is one thing that has improved communication between anaesthetists and surgeons*,* and that is the WHO checklist*,*… but at the beginning it’s the WHO checklist*,* where there are three periods of dialogue*,* pre-induction*,* pre-incision and pre-awakening*,* and normally in an ideal world*,* but the world often tends towards the ideal*,* it is at this point that the anaesthetists and surgeons talk to each other about “do you need antibiotics given what you are going to do*,* should they be continued in the postoperative period or not*,* etc.?* {Subject 25, Anaesthetist}
	Difficulties to stay up to date	Q13. *You have to stay up to date*,* you have to have a scientific watch on the subjects that interest you or on the subjects that are common in daily practice*,* and read the scientific data*,* interpret them according to their value*,* and apply that to your daily life*,* and so that’s the main obstacle* {Subject 16, Surgeon}
	Ignorance on the availability of SSI guidelines	Q14. *So specific reference material*,* no*,* apart from the surgical antibiotic prophylaxis guidelines*,* there is no real… I don’t think it’s clearly written that it’s for the prevention of SSI*,* I don’t know of any guidelines for the prevention of SSI* {Subject 21, Anaesthetist}
A collective ownership but a blame on the surgeon	Collective ownership but responsibility for surgeon	Q15. *The responsibility still lies with the anaesthetist and the surgeon*,* but perhaps a little more with the surgeon who feels more responsible.* {Subject 7, Anaesthetist}
	Failure for surgeons but error for anaesthetists	Q16. *Then the surgeon doesn’t have the same vision of the surgical site infection*,* he often sees it as a personal failure*,* a challenge*,* more than the anaesthetist.* {Subject 7, Anaesthetist}
	Lack of feedback of SSI leading to underestimations	Q17. *In my opinion*,* surgical site infections are underestimated […] Officially*,* the incidence is from 1 to 3% […] but we have mostly those that require hospital treatment in the battle.* {Subject 30, Anaesthetist}
	SAP relies on anaesthetists	Q18. *In any case*,* I think that surgical antibiotic prophylaxis is… it’s us who administer it*,* so we’re already responsible for it*,* we have to worry about it*,* we have to do it*,* we’re asked if we’ve done it or not*,* so there you go… for reinjections it’s up to us to think about it too*,* the surgeon*,* well*,* generally speaking*,* he trusts us relatively much.* {Subject 1, Anaesthetist}
Action focused surgeon and systemic oriented anaesthetists, leading to a positive cooperation	Positive experience in cooperation	Q19. *But we have very good coordination between the anaesthetists and the surgeons*,* so that’s not a problem* {Subject 28, Anaesthetist}.
	Need for multidisciplinary exchanges	Q20. *The spread of recommendations from anaesthetists to physicians*,* to surgeons*,* is often lacking […] and that is why MMRs and multidisciplinary preoperative meetings are essential for me.* {Subject 18, Surgeon}
	An interprofessional trust	Q21. *Uh*,* in our centre it’s excellent*,* no particular concerns. We have mutual trust*,* so they let us manage the prescription/administration of surgical antibiotic prophylaxis.* {Subject 31, Anaesthetist}
	Different approaches to guidelines across specialties	Q22. *No*,* I don’t think we would have any difficulties*,* because we adapt*,* I think that in anaesthesia we have a capacity for adaptation that is quite rapid*,* […] and I think that we apply them as soon as a recommendation is modified.* {Subject 26, Anaesthetist}
	Surgeons focused on immediate actions	Q23. *Let’s say that we think about the bleeding that is in front of us*,* we don’t necessarily think about the wall abscess that will be there in 2 weeks or in 8 days […] Because as surgeons*,* we work rather in action*,* it’s action-reaction*,* we don’t necessarily think*,* we don’t necessarily think about what’s going to happen 10 days later.* {Subject 6, Surgeon}
	Progressive withdrawal of anaesthetists	Q24. *It’s true that we are not… At one time*,* we were much more involved in postoperative care*,* but due to a staffing problem*,* we are less involved in the management of postoperative surgical site infections.* {Subject 33, Anaesthetist}
Lack of staff and heavy workloads, gravediggers of good wills	Work intensity	Q25. *In France we are the specialists to have to many irons in the fire… We are on call while we do consultations*,* we do intensive care while we are in the operating room. Well*,* it’s a little bit of everything… The other anaesthetists when they see us working they say “but you are crazy!* {Subject 27, Anaesthetist}
	Lack of staff resources	Q26. *There is a problem due to the important turnover in staff*,* leading to the need for constant training*,* staying alert. […] as soon as we start to change the teams in a very important way*,* we find ourselves exposed to much more frequent infections or problems of forgotten compresses or things like that.* {Subject 18, Surgeon}
	Difficult to comply with SAP timing	Q27. *Now it’s true that we perfuse them*,* we give them the antibiotic but it’s often in the operating room*,* so… I try to do it in orthopaedics*,* really*,* an hour before*,* but it’s not… it’s sometimes complicated. I think that we’re not… we’re a little short on that.* {Subject 1, Anaesthetist}
	Critical communication between each specialty	Q28. *Dialogue! Dialogue*,* having staff meetings together on complicated patients who have a problem during the consultation*,* sending an e-mail to the surgeon saying “your patient poses such and such a problem*,* I’m asking this*,* what do you think…”. Finally*,* the dialogue. Dialogue and discussing patients together.* {Subject 20, Surgeon}

Abbreviations: SSI, surgical site infection; SAP, surgical antibiotic prophylaxis

Ah, it is crucial because it concerns, after the success of the surgical procedure itself, the success of the anesthesia and the basic safety of the patient. It is the primary issue that will arise in the patient’s complaint and the follow-up of the patient… {Subject 34, Anesthetist}

Surgeons and anesthetists agreed that the digestive system is a special type of surgery, mainly because of the contaminated nature of these procedures occurring in an infected environment (Table 3, Q4). This led many participants to consider SSI in this field inevitable, even when fully complying with the guidelines (Table 3, Q5). They also shared a common perception of the consequences of SSI in digestive surgery, mostly associated with morbidity and the need for reoperation (Table 3, Q3). Among these consequences, antimicrobial resistance (AMR) has been frequently reported by both surgeons and anesthetists (Table 2, Q6).

…and then there’s an infection requiring bacterial sampling and then to antibiotics, and then to the emergence of resistance… {Subject 12, Surgeon}

While the overall importance of SSI was shared by surgeons and anesthetists, surgeons were more inclined to differentiate between deep infection and superficial infection (mainly wall abscesses) since the latter were seen to have lighter consequences and are easy to treat (Table 3, Q7).

Well, let’s say that if it’s a wall infection, it’s home care, so it’s something that doesn’t impact the patient too much, but if it’s a deep infection, yes, it’s repeat surgery, it’s longer hospital stays… {Subject 20, Surgeon}

Although the majority considered SSI to be important for the patient and the success of surgery, some reported that SSI prevention was not a priority in their daily tasks and was sometimes omitted by practitioners in favor of other important tasks expected from surgeons and anesthetists (Table 3, Q8).

I think that under the mass of work, the rhythm of the work… in the end, it is kind of omitted if you know what I mean […] Surgical site infection… We can all be responsible, so in the end, it gets diluted and we have the feeling that the responsibility… and therefore the awareness can be diluted in fact… {Subject 10, Anesthetist}

Many participants also reported ignoring the SSI rates in their departments, hospitals, and at the national scale.

In the 8 years I’ve been a department head, I have no idea what the department’s surgical site infections are. I have no idea at all. {Subject 6, Surgeon}

### A global trust in guidelines but an apparent knowledge gap

The vast majority of participants reported trusting producers of SSI prevention guidelines. This trust had a determining role in their willingness to apply for daily use. In general, they also considered the guidelines to be quite easy to follow (Table 3, Q10-11).

Yes, it is easy. Yes, yes it is easy because these measures are practical, well pragmatic, they are quantifiable, they are communicated to the entire French medical community, to my entire team… {Subject 32, Anesthetist}

The WHO surgical safety checklist was identified by participants as a key moment in surgery, allowing an exchange of information between surgical and anesthetist teams that might otherwise have been missed (Table 3, Q12). The difficulty for practitioners to stay up-to-date with guidelines was the most cited barrier to compliance. The multiplications of guidelines from different sources and sometimes contradictory recommendations, especially for skin preparation, were perceived as barriers to their use (Table 3Q13). Furthermore, a knowledge gap appeared in some participants, mainly surgeons, who did not know the sources of guidelines or used expired guidelines in daily use (Table 3, Q14).

I don’t have any knowledge… I mean… I use the knowledge I have and the… and what is done and what has been agreed in the operating room in terms of prevention, after which I have no… knowing if it’s the SFAR or if it’s some other company that does… Uh… no. {Subject 11, Surgeon}

### A collective ownership but blame on the surgeon

When questioned on the ownership of SSI, most participants stated that both anesthetists and surgeons were responsible for SSI prevention. However, when asked specifically about the responsibility for SSI, both surgeons and anesthetists conceded that surgeons were accountable for SSI (Table 3, Q15). Many reported that, since the surgeon was responsible for the recruitment of patients, he was expected to be mainly responsible for the entire process. Multiple anesthetists contributed to this, and only one surgeon intervened in patient care.

It’s still the surgeon-anesthetist pair, until proven otherwise. And then in the end, especially the surgeon (laughs) {Subject 11, Surgeon}

The sense of ownership among surgeons is also related to the notion of error. Many reported that SSI was the result of an error from the surgeon or team (Table 3, Q16).

The surgeons feel responsible if they are told that there is an infection of the operating site, they feel responsible for this surgical site infection even if they have nothing to do with it, and even if we had done everything possible according to the guidelines and prevent it as much as possible if it happens, I think it will happen. {Subject 21, Anesthetist}

Some even considered that the term of “surgical site infection” itself was pointing towards surgical teams. These factors are also believed to contribute to the endemic underreporting of SSI (Table 3, Q17).

It’s very often misclassified, I think… It’s under-used and under-valued because it’s a term that I think for our surgeons seems extremely pejorative as if they had a flaw in their interventions, a complication that would be their responsibility that they wouldn’t dare admit to,… I think they don’t want to hear it {Subject_21, Anesthetist}

**Fig. 1:**
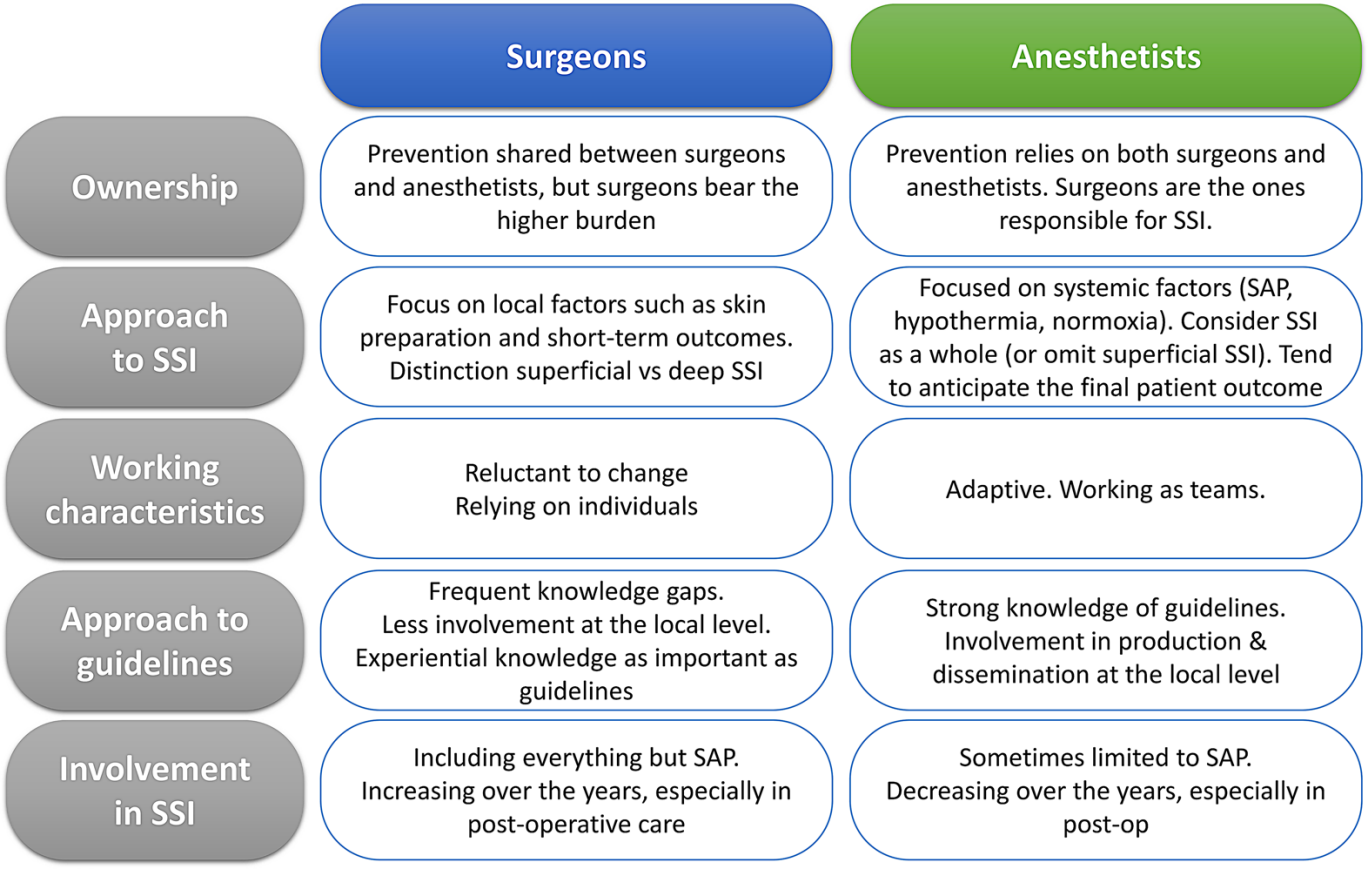
Mapping of cultural differences between surgeons and anesthetists

Ownership sometimes relies on anesthetists, especially for SAP. Some participants considered that surgeons were supposed to be involved in SAP, at the very least by asking about it based on the surgical safety checklist, but the majority believed SAP to be the anesthetist’s responsibility (Table 2, Q18). (Fig. 1)

### A positive Cooperation mixing surgeons focusing on tasks and anesthetists with holistic approaches

The majority of participants reported having a positive personal experience regarding cooperation between surgeons and anesthetists in the OR. Even if they had experienced difficulties or had heard of difficulties at some point, they were generally believed to be mainly related to individual factors (Table 3, Q19). Participants insisted on the need for multidisciplinary exchanges between surgeons and anesthetists during pre-, per-, and postoperative care. (Table 3, Q20) Participants highlighted the importance of trust in their colleagues and the necessity for each participant to know each other before the surgical procedure (Table 3, Q21). Surgeons and anesthetists also felt that they had different approaches to the guidelines. Anesthetists have been reported to be involved in guideline production and diffusion across professional societies and hospitals (Table 3, Q22).

The anesthetists are perhaps less present in front of the patient because they have a lot of things to do,… I find them very involved because I have many surgical colleagues who are always attacking them… Frankly, they are on all the committees, they monitor good practices, and I think that’s essential. {Subject 14, Surgeon}

Anesthetists are structured to work as teams, with several physicians involved in each step of care. This organization does not impair the quality of care because anesthetists are accustomed to following guidelines, minimizing the effects of individual behaviors.

The anesthetist sees the patient pre-operatively alone without discussing with the surgeon, the case is seen at the staff meeting with the presence of an anesthetist, sometimes not the one who manages the patient afterwards, you know that the anesthetists rotate a lot… {Subject 16, Surgeon}

However, surgeons have been reported to be less involved in guideline production. Their practices rely mainly on their experiences inherited from training and practices. As such, the quality of care was carried out by individuals who were not easily replaced. They were also described as more reluctant to change their work habits.

… you can have clearer, more outspoken opposition from the surgeon, who tends to want to remain in a routine… The neurosurgeon who has been shaving heads for 30 years, giving his patient 4 showers, 4 shampoos…, and who does the scrubbing himself… You won’t be able to change this. {Subject 14, Surgeon}

Anesthetists are considered to be involved in the systemic determinants of SSI, such as hypothermia, oxygenation, nutrition, and blood pressure. Surgeons described themselves as more focused on the surgical site, focusing mainly on direct risk factors, such as skin preparation, and sometimes omitting other risks (Table 3, Q23). Participants reported a declining role for anesthetists in SSI, especially in postoperative care, mainly due to a shortage of staff and an expansion of their missions (Table 2, Q24).

### Lack of staff and heavy workloads, gravediggers of good wills

Many participants declared workload as one of the main barriers to applying SSI preventive measures. In both the public and private sectors, this intensity leads to omitting some tasks, either deliberately to gain time during procedures or unconsciously forgetting one step due to the heavy workload (Table 3, Q25).

So the amount of work and the working conditions definitely influence the aftermath of a patient’s surgery, whether it’s in the form of infection or otherwise. {Subject 16, Surgeon}

The lack of both medical and nursing staff led to an important turnover of professionals in the OR and gaps in education and training (Table 3, Q26). Heavy workload and outpatient surgery were often identified as barriers to SAP timing (Table 3, Q27). Another barrier included breaches in the communication between specialties, which are considered to be central to SSI prevention (Table 3, Q28). Suboptimal communication was not due to the physicians themselves, but rather to organizational factors such as the busy schedule of anesthetists.

## Discussion

Although considered an important issue by surgeons and anesthetists, SSIs were perceived as an inevitable consequence of digestive surgery, but were not viewed as a top priority in their routine. The perception of SSI by digestive surgeons as easy to treat with low individual burden has been previously described [4]. Growing evidence regarding the efficiency of preventive measures in digestive surgery (up to -50% of SSI in large bowel surgery) challenges the view that these infections are generally not preventable [19–21]. Finally, surgeons tended to discriminate superficial infections as not of clinical importance, while deep infections represented a major complication.

The participants were concerned about the burden of SSI. However, a distinction was made between preventable factors belonging to the organisation/resources, and those perceived as beyond the control of the surgical team such as the wound contamination. Wound contamination in digestive surgery has traditionally been viewed as difficult to avoid, which has contributed to less intensive efforts toward SSI prevention [4]. The available evidence on the effectiveness of prevention bundles in colorectal surgery supports the notion that SSIs are avoidable, even in high-risk procedures [22]. In England, large bowel surgery, together with caesarean section, is the highest contributor to total SSIs, with 39,000 (38%) cases annually [2]. However, few surgeons or anesthetists are informed of SSI incidence in their hospitals or countries. This finding confirms the need to improve feedback on patient outcomes to professionals [23–26]. This feedback leads to a reduction in SSI rates and an increase in compliance when associated with other infection-control practices [27]. Although a national SSI surveillance system exists for digestive surgery in France, the methods used by stakeholders for sharing the results with frontline staff remain insufficient [28]. Beyond the importance of SSI, our study suggests that surgeons and anesthetists perceive the risk of AMR as a threat that affects the outcomes of patients undergoing digestive surgery. The mention of AMR by many participants suggests a high awareness in this field. In recent years, studies have highlighted the burden of AMR, especially in low resources countries [29]. Even in high-income countries, SSI caused by resistant organisms is already a burden responsible for high morbidity [30], which could eventually question the risk-benefit balance of digestive surgery and represent an obstacle for surgery in the future [31].

One of the most striking results was related to ownership and participants’ perceptions of the respective roles of surgeons and anesthetists in SSI. Although the responsibility for SSI was mostly perceived as collective, surgeons were considered responsible. Some even stated that the individual nature of the surgery, with the surgeon in charge of the patient across the perioperative pathway, was the reason for such a responsibility. Anesthesia is administered by several anesthetists throughout the patient’s pathway rather than by a single person identified to the patient. Ownership and responsibility are also linked to the notion of mistakes in the surgeon’s mind. While many anesthetists consider the occurrence of SSI as the natural course of surgery, surgeons are more inclined to feel guilty about the infection. Some participants even suggested that the denomination “SSI” pointed directly to the surgeon and was perceived by the surgeons as a question of their performance. Previous studies have described the emotional impact of SSI on surgeons and its association with direct guilt and responsibility [32–34], but few have compared this feeling with that of anesthetists. SSI prevention is a shared responsibility that involves the entire surgical team throughout the surgical pathway. Consequently, ownership of SSI prevention and accountability for outcomes should be distributed across all staff categories and not only surgeons, beginning with accurate outcome reporting.

The overarching culture differs between the two specialties. Surgeons described themselves as focused on immediate tasks, sometimes favoring short-term outcomes, whereas anesthetists tended to anticipate long-term outcomes [4, 35, 36]. Regarding guidelines, anesthetists were all able to name the scientific society responsible for SAP guidelines and almost all knew the details of the local protocol in their institutions, which was not the case for surgeons. Surgery seems to be based on individual practice and knowledge, with skills being acquired more empirically by senior to junior surgeons [37]. Anesthetists appear to acquire their knowledge and skills from collective transmission through guidelines and literature. The collective organization of the work of anesthetists as a team with interchangeable individuals mandates this culture. The contrast between the norm of individualism promoted by surgeons (where one physician decides with little input from others) and the collectivism of internal physicians has already been described in antimicrobial stewardship [38]. Consequently, surgeons were considered more reluctant to evolve guidelines than anesthetists, for whom a new guideline seemed to be easily accepted and implemented. While identical levels of knowledge are not required for tasks specific to surgeons or anesthetists, a cross-specialty minimum knowledge base should be established, along with awareness of existing guidelines. The collaborative approach commonly seen among anesthetists may serve as a valuable model for surgeons. Encouraging commitment could offer promising ways to address surgeons’ reluctance to engage in the development of clinical guidelines.

Organizational factors have also been identified as key drivers of SSI prevention [39]. In 2012, 59% of all reported adverse events involved the relationship between anesthetists and surgeons along the surgical path: preoperative (26%), intraoperative (22%), and postoperative (39%) [40]. In our study, both anesthetists and surgeons highlighted the importance of trust in their colleagues and the necessity for each participant to know each other before the surgical procedure. This finding is in line with that of a retrospective study that examined the association between surgeons’ and anesthetist familiarity with patient outcomes. The authors found that increasing familiarity with the surgeon-anesthetist dyad was associated with improved short-term outcomes in patients undergoing digestive surgery [41]. The latest is challenged by new operating platforms organized with specialties that mutualize multiple rooms and staff. Prioritizing efforts on organizational aspects, optimization of teamwork, and cooperation are key to enhancing surgical performance by enabling early issue detection, reducing practice breaches, ensuring consistency and mutual oversight, and fostering collective learning and continuous improvement [42]. As key players, surgeons must be actively engaged—their involvement enhances clinical leadership, drives change, and motivates the entire team.

The main limitation of this study was its focus on physicians and excluded non-medical staff. Non-medical staff such as operating room nurses are strongly contributing to the prevention of SSI. However, we chose to focus on physician for its involvement in guideline production and the leading role of physicians poorly explored in digestive surgical teams. Second, interviews were conducted by an investigator trained in IPC, which could have led to declaration bias. Finally, we anticipated that participants’ responses would be influenced by their experience or the sector in which they worked; however, we did not find significant differences when analyzing the interviews.

## Conclusion

Although perceived as an inevitable complication in digestive surgery, SSI should become a top priority for patient safety and AMR prevention in this specialty. The cultural differences between surgeons and anesthetists are critical to understanding the ownership and responsibilities of SSI, improving teamwork and cooperation, supporting the efficient use of guidelines, and better complying with preventive measures.

## Electronic supplementary material

Below is the link to the electronic supplementary material.

Supplementary Material 1

## Data Availability

No datasets were generated or analysed during the current study.
