# Supplementary material for: Cultural differences in attitudes towards surgical site infections among French anesthetists and surgeons in digestive surgery in 2022
Source: Antimicrob Resist Infect Control. 2025 May 28;14:57. doi: 10.1186/s13756-025-01576-9 (PMC12121121; doi:10.1186/s13756-025-01576-9)
Supplement: Supplementary file 1 — Supplementary Material 1 [file 13756_2025_1576_MOESM1_ESM.docx]

#### **Supplementary materials**

#### **Supplementary Table 1. Interviews – Coding framework**

| **Theme** | **Code** |
| --- | --- |
| SSI representations | Consequences can be dire |
|  | SSI are inevitable in digestive surgery |
|  | Hard to lower incidence more |
|  | SSI is not a priority |
|  | Particular brand of surgery |
| Ownership | Shared ownership between surgeon and anesthetists |
|  | Surgeon is responsible |
|  | Anesthetist is responsible for antimicrobial prophylaxis |
|  | Guilt feeling |
| Guidelines perception | Knowledge gap |
|  | Guidelines can be futile |
|  | Lack of application |
|  | Need for more control |
|  | Multiple guidelines can be a problem |
|  | Hard to stay on top of guidelines |
| Imperfect cooperation between anesthetist and surgeon | Need for a leader |
|  | Personal relations influence cooperation and SSI prevention |
|  | Room for improvement in cooperation |
|  | Roles are sometimes unclear |
|  | Egos can be problematic |

#### **Supplementary Table 2. Interviews – Original quotes in French**

| **Quote identification** | **Original quote** |
| --- | --- |
| V1 | ah c'est primordial parce que c'est ce qui va concerner quand même, après la réussite du geste chirurgical en soit, et la réussite de l'anesthésie et la sécurité du patient on va dire de base, c'est la première problématique qui va survenir dans la réclamation du patient donc... Et dans le suivi du patient donc... Donc c'est quand même primordial c'est... c'est l'équivalent de la réussite d'un geste opératoire c'est l'équivalent de... du... de la procédure anesthésique |
| V2 | et puis ensuite il y a un problème infectieux et infectiologique c'est à dire que ça peut être un recours au prélèvement bacterio puis aux antibiotiques, puis à l'émergence de résistance qui font qu'il y a une relation entre le médical et l'économie |
| V3 | Ben disons que si c'est une infection de paroi c'est des soins à la maison donc c'est quelque chose qui n'impacte pas trop trop le patient, après si c'est une infection profonde, oui c'est des reprises chirurgicales, c'est des allongements de la durée d'hospitalisation.. |
| V4 | Parce que, parce que... Je pense que sous la masse de travail, le rythme du travail.. Bah en fait c'est un peu passé à l'as, si vous voyez ce que je veux dire […] L'infection du site opératoire on peut tellement être tous responsables que en fin de compte ça se dilue et on a la sensation que la responsabilité et donc la sensibilisation peut se diluer en fait… Trop d'autres choses à penser. |
| V5 | Depuis 8 ans que je suis chef de service et bah j'ai aucune idée des infections du site opératoire du service. Je ne sais pas du tout. |
| V6 | Oui c'est facile. Oui, oui c'est facile parce que ces mesures sont pratiques, enfin pragmatiques, elles sont quantifiables, elles sont communiquées à l'ensemble de la communauté médicale française, à l'ensemble de mon équipe… |
| V7 | J'ai aucune connaissance... Enfin je veux dire.. J'utilise les connaissances que j'ai eu et les... et ce qui se fait et qui a été acté au bloc opératoire dans la prévention, après j'ai pas de... savoir si c'est la SFAR ou si c'est telle autre société qui fait... Euh... non. |
| V8 | ça reste quand même le couple chirurgien anesthésiste quand même hein, jusqu’à preuve du contraire. Et puis in fine surtout le chirurgie (rires) |
| V9 | Les chirurgiens eux se sentent responsables si on leur dit qu'il y a une infection du site opératoire, ils se sentent responsables de cette infection du site opératoire même si ils n'y sont pour rien, et on aurait eu beau tout faire être parfaitement dans l'écoute des guidelines et la prévenir au maximum si elle arrive je pense qu'elle arrivera. |
| V10 | C'est très souvent mal classé, je pense que c'est sous... Le terme est sous utilisé et sous évalué parce que c'est un terme qui je pense pour nos chirurgiens paraît extrêmement péjoratif comme si ils avaient eu une faille dans leurs interventions, une complication qui serait de leur propre responsabilité qu'ils n'oseraient pas s'avouer et donc je pense que c'est sous-évalué et que c'est un problème bien plus important que ce qu'on ne pense, mais juste sous-évalué rien que déjà sur l'utilisation du terme et sur ce qu'il sous-entend où il a un aspect péjoratif pour nos chirurgiens comparativement on s'en fout quoi, il y a des ISO, ça existe et voilà mais... Je pense qu'ils ne veulent pas l'entendre. |
| V11 | es anesthésistes, ils sont peut-être moins présents en face du malade parce qu'ils ont beaucoup de choses à faire mais par contre sur ces questions-là moi je les trouve très impliqués, parce que j'ai beaucoup de collègues chirurgiens qui sont tout le temps en train de s'en prendre à eux, ils seraient pas là on serait pas dans la même situation, franchement ils sont dans toutes les commissions ils veillent quand même sur les bonnes pratiques, c'est quand même essentiel hein je pense.. |
| V12 | l'anesthésiste voit le malade en préopératoire seul sans discuter avec le chirurgien, le dossier est vu au staff avec présence des anesthésistes parfois ce ne sont pas eux qui gèrent les malades après, vous savez qu'ils tournent beaucoup les anesthésistes |
| V13 | vous pouvez avoir par contre des oppositions plus nettes, plus marquées de la part du chirurgien qui lui a tendance à vouloir rester dans une routine voilà le neurochir ça fait 30 ans qu'il rase la tête, qu'il fait 4 douches, 4 shampoing à la bétadine à son patient, il fait lui-même le badigeon vous allez pas le faire changer en fait. |
| V14 | Donc la masse de travail et les conditions de travail influent de manière certaine sur les suites opératoires d'un malade opéré, qu'elles soient sous forme de d'infection ou autres. |
| Q1 | Ah bah quand on fait de la chirurgie digestive c'est majeur parce que c'est quand même un des... Un des gros problèmes post opératoire de cette chirurgie.. […} Non non ça fait partie intégrante de la spécialité |
| Q2 | Alors les conséquences ça va du simple au très compliqué hein, sachant que le très compliquée souvent très très lourd dans cette spécialité donc euh voilà.. |
| Q3 | Alors comme on opère un organe qui est plein de microbes, donc les infections du site opératoire sont majeures et sont plus importantes que dans d'autres spécialités telles que la chirurgie orthopédique, telles que la neurochirurgie |
| Q4 | on travaille en milieu septique, et certaines infections arrivent même en respectant les mesures. On est sur le tube digestif qui est un organe "nul", il a tendance assez facilement à déverser son contenu et créer des infections |
| Q5 | un malade sur 5, va présenter une infection de site opératoire c'est un problème qui va... qui va entraîner une surmorbidité, qui va entraîner une prolongation du séjour à l'hôpital, qui va entraîner des séquelles au niveau de la paroi avec un risque notamment d'éventration etcaetera, un retard de convalescence, et et un coût pour la société |
| Q6 | Très clairement on voit en France un contexte de surutilisation des antibiotiques, d'émergence de bactéries résistantes, et de nécessité pourtant d'utilisation de ces antibiotiques pour prévenir les infections du site opératoire, donc bah il est très important pour moi dans le contexte français d'avoir une aide à la mise en œuvre d'une antibioprophylaxie adaptée lors de mes gestes anesthésiques |
| Q7 | Donc la chirurgie viscérale et digestive et très large, il faut séparer deux situations enfin plusieurs situations qui sont à risque : il y en a une qui te rejoint celle de l'orthopédie c'est la chirurgie de paroi, où là en fait nous n'avons pas normalement de contamination du du du site opératoire mais avec l'implantation d'une prothèse le risque infectieux.. c'est pas qui qu'il est augmenté c'est qu'il est plus difficile à gérer |
| Q8 | Je pense que sous la masse de travail, le rythme du travail.. Bah en fait c'est un peu passé à l'as, si vous voyez ce que je veux dire […] Trop d'autres choses à penser. |
| Q9 | Je pense que, enfin en tout cas ce qu'on nous transmets comme information, qu'on n'a pas d'ailleurs c'est regrettable, ce serait bien qu'on puisse avoir en fait, quasiment en temps réel les chiffres de ces infections de site opératoire |
| Q10 | Non bah j'ai complètement confiance les recommandations elles sont... Elles vont .. Jai confiance en ma société savante et dans les recommandations qu'elle sort |
| Q11 | Bah ça ne me parait pas.., sur ce qu'on nous demande ça ne me parait pas insurmontable, c'est de la préparation, c'est des protocoles établis, après c'est de faire attention un peu.. […]donc ça ne me parait pas si compliqué que ça. |
| Q12 | Et puis il faut qu'on discute, donc ça c'est pas mal c'est une... ça se fait pas trop mal chez moi, mais il y a un truc qui a quand même amélioré la communication anesthésiste-chirurgien c'est la check-list de l'OMS, relayée par la HAS, mais au départ c'est la check-list OMS, où on prévoit trois temps de dialogue, en pré-induction, en pré-incision et en pré-réveil, et normalement dans un monde idéal mais le monde souvent tend vers l'idéal c'est à ce moment-là que les anesthésistes et chirurgiens se parlent sur "as-tu besoin d'antibiotiques compte tenu de ce que tu vas faire, est ce qu'il faut les poursuivre en post opératoire ou pas etc. |
| Q13 | il faut rester à jour, il faut avoir une veille scientifique sur les sujets qui nous intéressent ou es sujets courants de la pratique quotidienne, et lire les données scientifiques, les interpréter en fonction de leur valeur, et appliquer ça au quotidien, et donc c'est ça le principal frein |
| Q14 | Alors de référentiel spécifique non en dehors des guidelines antibioprophylaxie, il n'y a pas de vrai.. Je crois pas que ce soit écrit clairement que c'est en prévention des ISO, des guidelines de prévention des ISO à ma connaissance j'en connais pas comme ça |
| Q15 | La responsabilité repose encore sur l'anesthésiste et le chirurgien, mails peut être un peu plus sur le chirurgien qui se sent plus responsable |
| Q16 | Ensuite le chirurgien n'a pas la même vision de l'infection de site opératoire, il la voit souvent comme un échec personnel, une remise en cause, plus que l'anesthésiste |
| Q17 | les infections de site opératoire sont à mon avis sous-évaluées [...]Officiellement l'incidence elle est de 1 à 3 % […] mais on a surtout celles qui nécessitent un traitement hospitalier dans la bataille |
| Q18 | Ben toute façon l'antibioprophylaxie je pense que c'est... c'est nous qui l'administrons donc déjà on en est responsables, on doit s'en préoccuper, on doit la faire, on nous demande si on l'a faite ou pas donc voilà... pour les réinjections c'est à nous d'y penser aussi, le chirurgien enfin en général il nous fait relativement confiance hein |
| Q19 | mais nous on a une très bonne coordination entre les anesthésistes et les chirurgiens donc ça pose aucun problème. |
| Q20 | Les diffusion des recommandations des anesthésistes auprès des médecins, auprès des chirurgiens qui manquent souvent […] c'est pour ça que les RMM, les réunions multidisciplinaires de réunions préopératoires sont essentielles pour moi |
| Q21 | Euh dans notre centre c'est excellent, pas de soucis particuliers. On a une confiance mutuelle qui fait qu'ils nous laissent gérer la prescription/administration de l'antibioprophylaxie. |
| Q22 | Non je pense pas qu'on aurait des difficultés, parce que on s'adapte, on a je trouve en anesthésie une capacité d'adaptation qui est assez rapide […] et je pense qu'on met en application des qu'une recommandation est modifiée de façon assez rapide. |
| Q23 | Disons qu'on pense au saignement qui est devant nous, on ne pense pas forcément à l’abcès de paroi qu'il y aura dans 2 semaines ou dans 8 jours quoi […] Parce que les chirurgiens on travaille plutot dans l'action quoi, c'est action réaction on pense pas forcément, on ne réfléchit pas forcément à ce qui va se passer 10 jours après |
| Q24 | C'est vrai qu'on n'est pas.. A un moment on intervenait beaucoup plus dans la prise en charge post opératoire mais du fait d'un problème d'effectif là actuellement on le fait moins, on est quand même moins partie prenante dans la gestion des infections de site opératoire en post opératoire. |
| Q25 | en France on est les spécialistes de se retrouver au four et au moulin quoi... On est de garde pendant qu'on fait les consultations, on fait les soins intensifs pendant qu'on est au bloc opératoire.. Enfin c'est un petit peut tout ça... Les autres anesthésistes quand ils nous voient travailler ils nous disent "mais vous êtes fous vous!" |
| Q26 | Il y a une problématique de turnover encore de personnel qui fait que à bah à chaque fois faut les reformer et être vigilant [...] dès qu'on commence un peu à changer les équipes de manière très importante on se retrouve effectivement exposés à des infections beaucoup plus fréquentes ou des problématiques d'oubli de compresses ou de choses comme ça |
| Q27 | maintenant c'est vrai qu'on les perfuse on leur passe l'antibiotique mais c'est souvent en salle d'intervention donc euh.. j'essaie du coup en en orthopédie de le faire vraiment une heure avant mais c'est pas... c'est parfois compliqué. Je pense que là dessus on n'est pas... on est un peu justes |
| Q28 | Le dialogue, faire des staffs ensemble sur les malades compliqués, il sont une difficulté pendant la consultation, envoyer un mail au chirurgien en disant "voilà ton patient il pose tel problème, je demande ça, qu'est ce que tu en penses..." Enfin voilà, le dialogue. Le dialogue et discuter des patients ensemble. |

Footnotes: V1-V14 are quotes cited in the text of the manuscript. Q1-Q28 are similar to quotes from Table 3.

**Supplementary Table 3. Semi-structured interviews - Participants characteristics**

| **Participant** | **Occupation** | **Gender** | **Region** | **Facility** | **Face to face interview** | **Experience (years)** |
| --- | --- | --- | --- | --- | --- | --- |
| Subject 1 | Anesthesia | Female | Pays de Loire | Private, for profit hospital | Yes | 25 |
| Subject 2 | Anesthesia | Female | Pays de Loire | Private, for profit hospital | No | 28 |
| Subject 3 | Surgery | Female | Pays de Loire | Private, not for profit hospital | No | 8 |
| Subject 4 | Anesthesia | Female | Pays de Loire | Private, for profit hospital | No | 18 |
| Subject 5 | Anesthesia | Female | Pays de Loire | Public sector | No | 10 |
| Subject 6 | Surgery | Female | Pays de Loire | Public sector | No | 18 |
| Subject 7 | Anesthesia | Female | Pays de Loire | Private, not for profit hospital | No | 10 |
| Subject 8 | Anesthesia | Female | Pays de Loire | Public sector | No | 5 |
| Subject 9 | Surgery | Male | Centre Val de Loire | Private, for profit hospital | No | 29 |
| Subject 10 | Anesthesia | Female | Pays de Loire | Private, for profit hospital | No | 14 |
| Subject 11 | Surgery | Male | Pays de Loire | Private, for profit hospital | Yes | 10 |
| Subject 12 | Surgery | Female | Centre Val de Loire | Private, for profit hospital | No | 9 |
| Subject 13 | Surgery | Female | Auvergne Rhone Alpes | Private, for profit hospital | No | 11 |
| Subject 14 | Surgery | Male | Auvergne Rhone Alpes | Private, for profit hospital | No | 13 |
| Subject 15 | Surgery | Male | Hauts de France | Private, for profit hospital | No | 20 |
| Subject 16 | Surgery | Male | Auvergne Rhone Alpes | Public sector | No | 32 |
| Subject 17 | Surgery | Male | Ile de France | Public sector | No | 22 |
| Subject 18 | Surgery | Male | Occitanie | Private, for profit hospital | No | 25 |
| Subject 19 | Surgery | Female | Ile de France | Private, not for profit hospital | No | 19 |
| Subject 20 | Surgery | Female | Ile de France | Public sector | Yes | 25 |
| Subject 21 | Anesthesia | Male | Grand Est | Public sector | No | 6 |
| Subject 22 | Surgery | Male | Hauts de France | Public sector | No | 10 |
| Subject 23 | Surgery | Male | Pays de Loire | Private, for profit hospital | Yes | 17 |
| Subject 24 | Anesthesia | Male | PACA | Private, for profit hospital | No | 22 |
| Subject 25 | Anesthesia | Female | Ile de France | Private, not for profit hospital | No | 33 |
| Subject 26 | Anesthesia | Male | Auvergne Rhone Alpes | Private, for profit hospital | No | 3 |
| Subject 27 | Anesthesia | Male | Occitanie | Public sector | No | 18 |
| Subject 28 | Anesthesia | Female | PACA | Public sector | No | 15 |
| Subject 29 | Anesthesia | Male | Centre Val de Loire | Public sector | No | 20 |
| Subject 30 | Anesthesia | Female | Ile de France | Public sector | No | 9 |
| Subject 31 | Anesthesia | Male | PACA | Public sector | No | 4 |
| Subject 32 | Anesthesia | Male | Auvergne Rhone Alpes | Private, for profit hospital | No | 12 |
| Subject 33 | Anesthesia | Female | Occitanie | Public sector | No | 12 |
| Subject 34 | Anesthesia | Male | Ile de France | Private, for profit hospital | No | 16 |
